# Supplementary material for: Pseudogenes document protracted parallel regression of oral anatomy in myrmecophagous mammals
Source: Mol Biol Evol. 2026 Jan 13;43(2):msag009. doi: 10.1093/molbev/msag009 (PMC12906968; doi:10.1093/molbev/msag009)

Supplementary Figure S1. RAxML gene trees based on alignments after cleaning in preparation for PAML analyses.

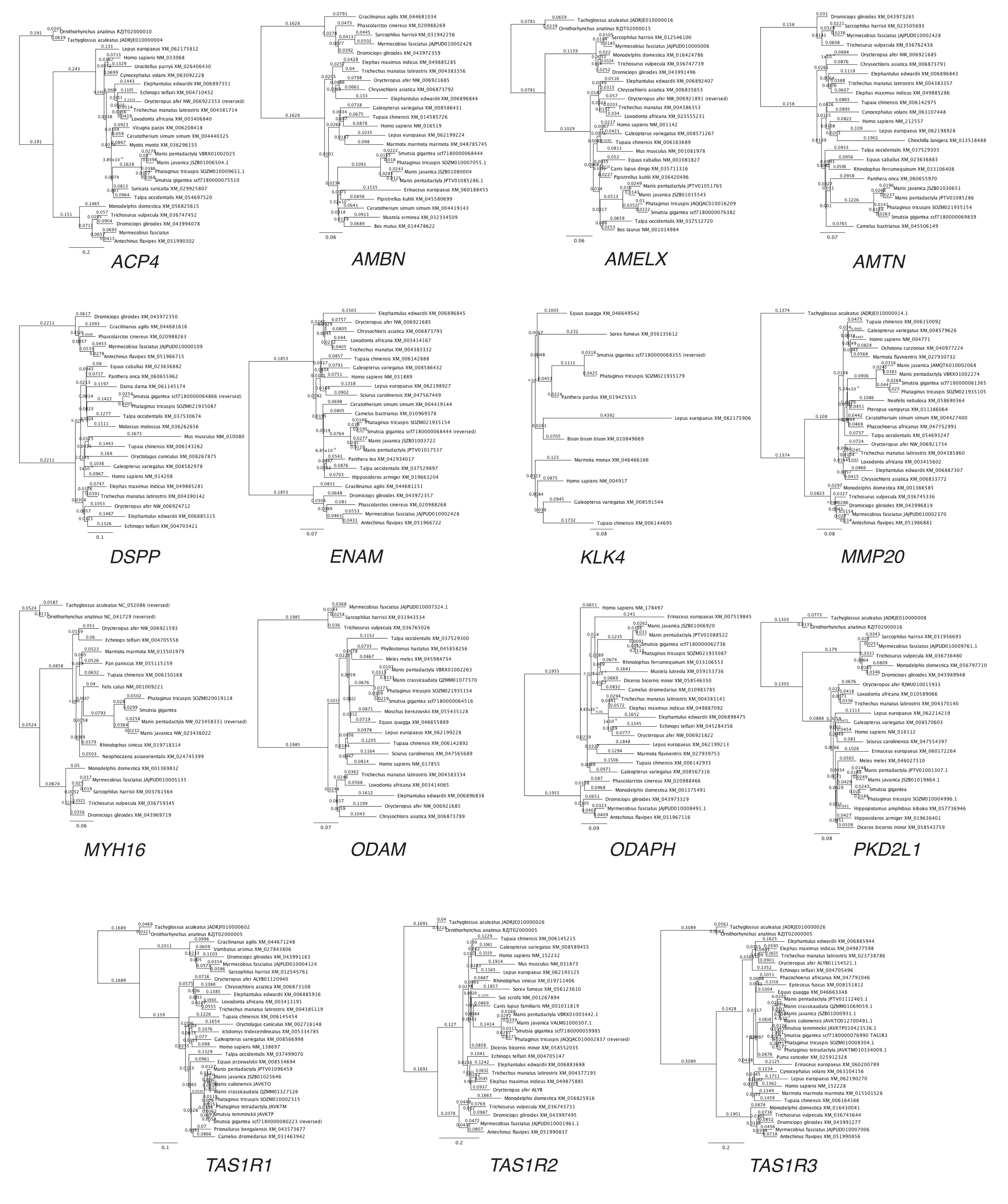

Supplement: msag009_Supplementary_Data [file msag009_supplementary_data.zip › Supplementary Figure S1. Gene Trees.pdf]
