# Supplementary material for: Pseudogenes document protracted parallel regression of oral anatomy in myrmecophagous mammals
Source: Mol Biol Evol. 2026 Jan 13;43(2):msag009. doi: 10.1093/molbev/msag009 (PMC12906968; doi:10.1093/molbev/msag009)

Supplementary Figure S12. DNA sequence alignments for monotreme genes. Gray annotations indicate coding exons in reference mRNAs. Yellow annotations for *MMP20* indicate predicted CDS on NCBI RefSeq for *Antechinus flavipes*. Pink annotations indicate inactivating mutations.

Monotreme *ACP4*

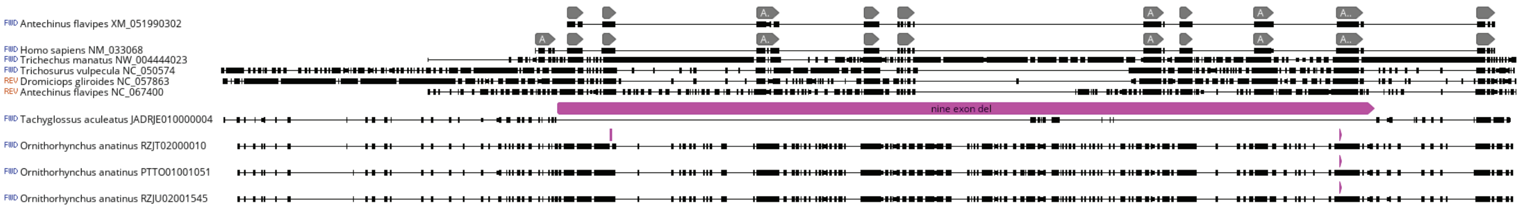

Monotreme *AMELX*

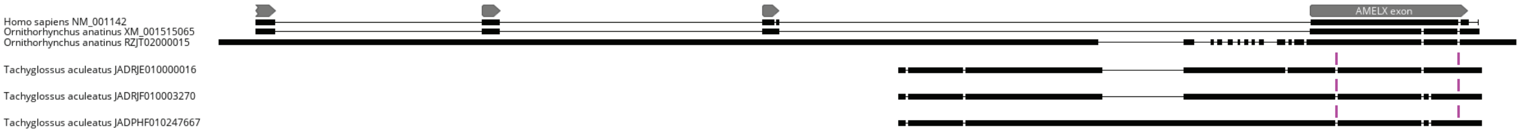

Monotreme *MYH16*

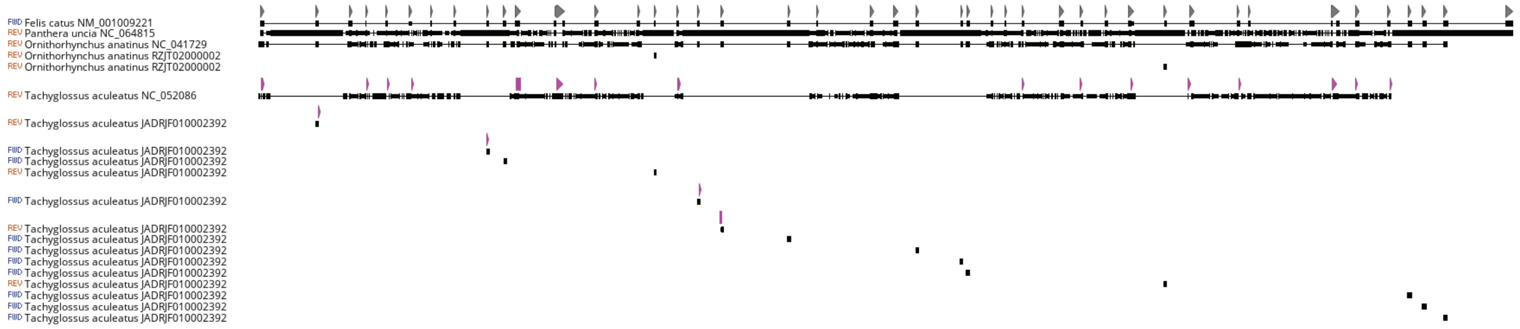

Monotreme *PKD2L1*

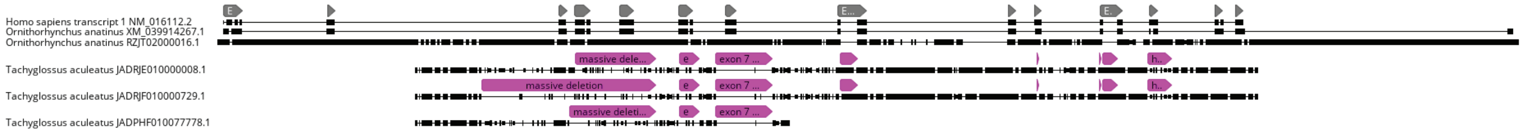

Echidna *MMP20*

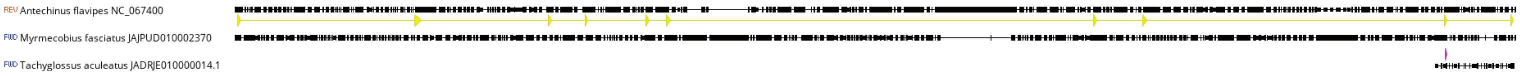

Supplement: msag009_Supplementary_Data [file msag009_supplementary_data.zip › Supplementary Figure S12. Monotreme Mutations.pdf]
