# Supplementary material for: Pseudogenes document protracted parallel regression of oral anatomy in myrmecophagous mammals
Source: Mol Biol Evol. 2026 Jan 13;43(2):msag009. doi: 10.1093/molbev/msag009 (PMC12906968; doi:10.1093/molbev/msag009)

Supplementary Figure S3. DNA sequence alignments for ostentorian (Carnivora + Pholidota) genes. Gray annotations indicate coding exons in reference mRNAs. Pink annotations indicate inactivating mutations.

Ostentoria *DSPP*

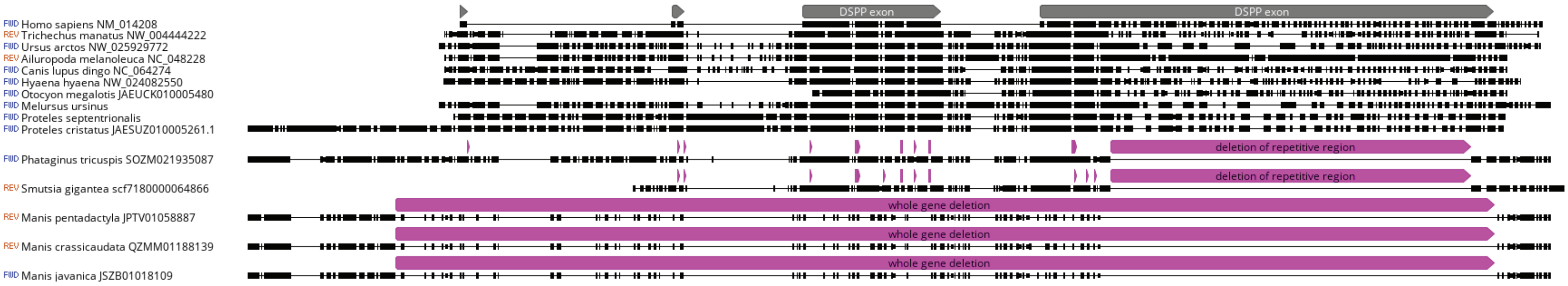

Ostentoria *ENAM*

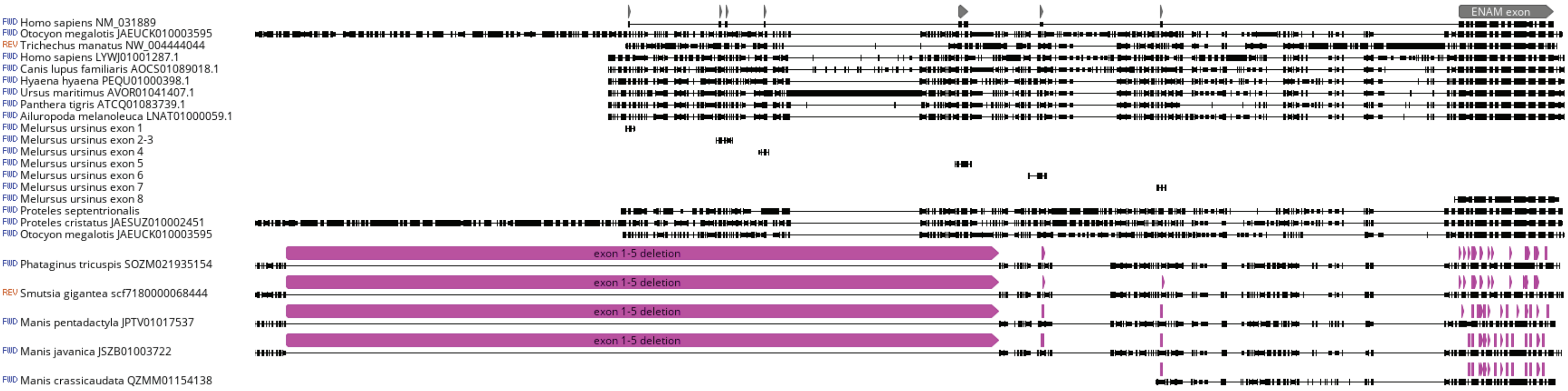

Ostentoria *KLK4*

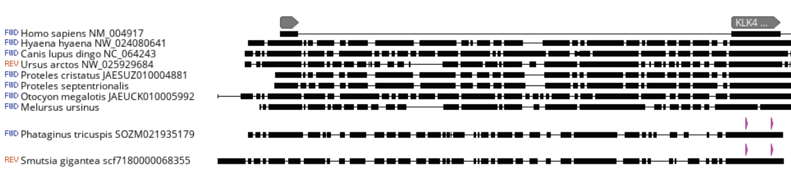

Ostentoria *MMP20*

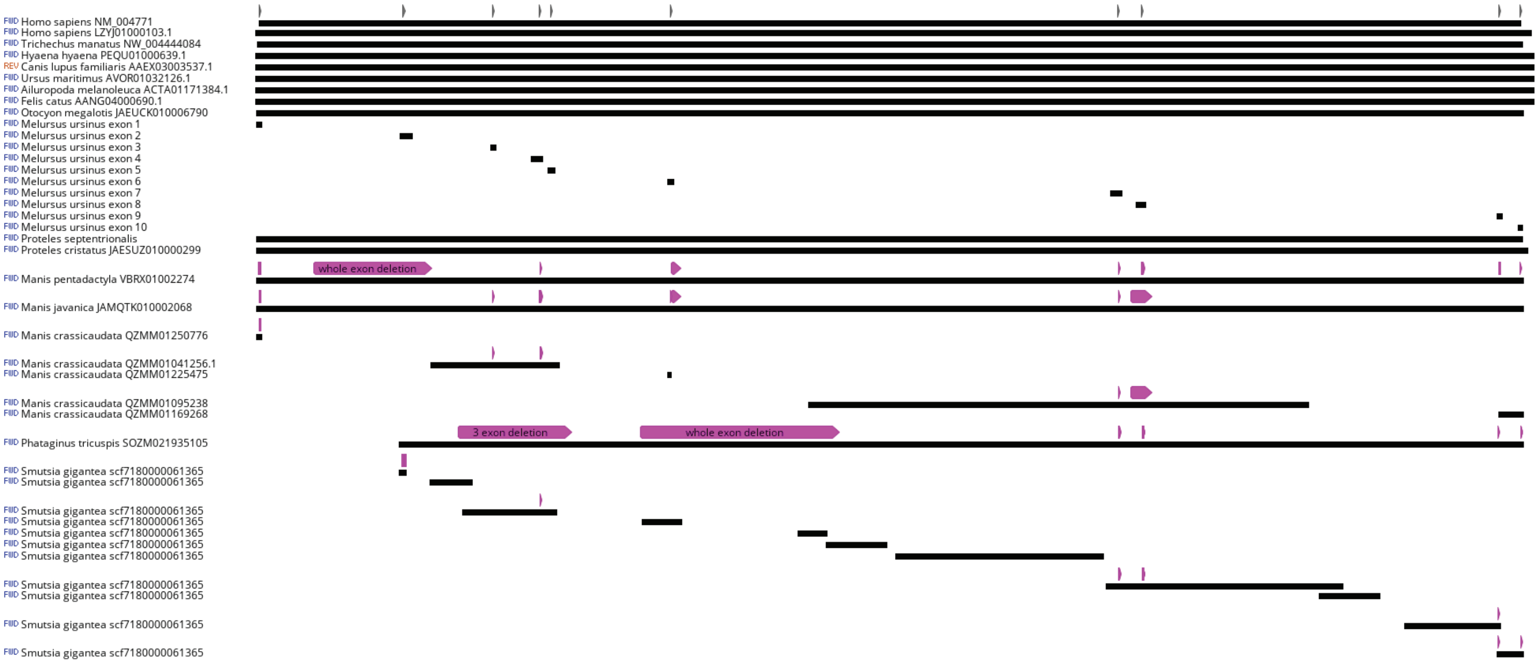

Supplement: msag009_Supplementary_Data [file msag009_supplementary_data.zip › Supplementary Figure S3. Ostentoria DSPP ENAM KLK4 MMP20.pdf]
