# Supplementary material for: Pseudogenes document protracted parallel regression of oral anatomy in myrmecophagous mammals
Source: Mol Biol Evol. 2026 Jan 13;43(2):msag009. doi: 10.1093/molbev/msag009 (PMC12906968; doi:10.1093/molbev/msag009)

Supplementary Figure S4. DNA sequence alignments for ostentorian (Carnivora + Pholidota) genes. Gray annotations indicate coding exons in reference mRNAs. Pink annotations indicate inactivating mutations.

Ostentoria *ODAM*

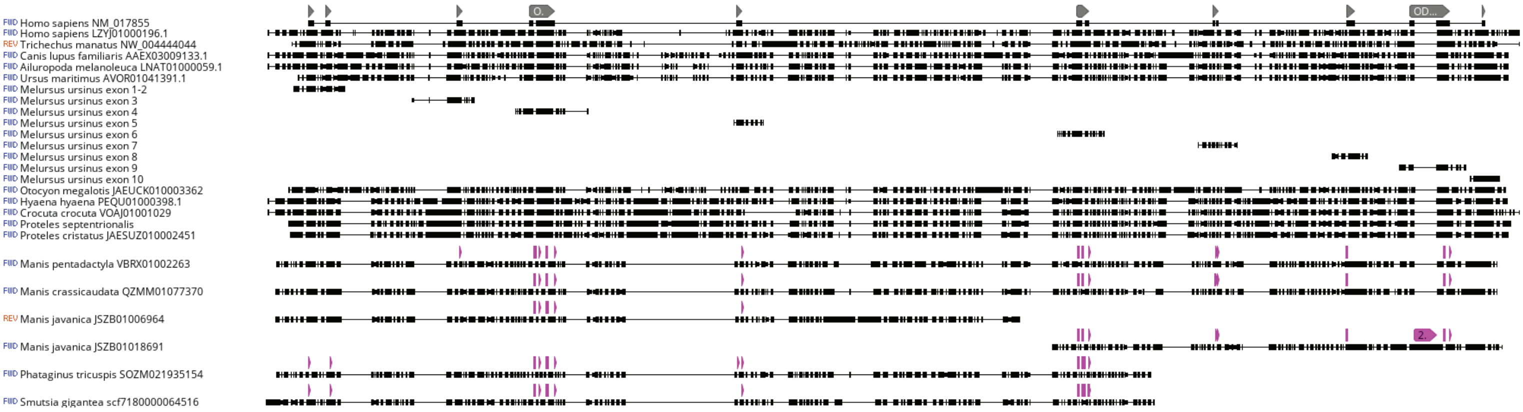

Ostentoria *ODAPH*

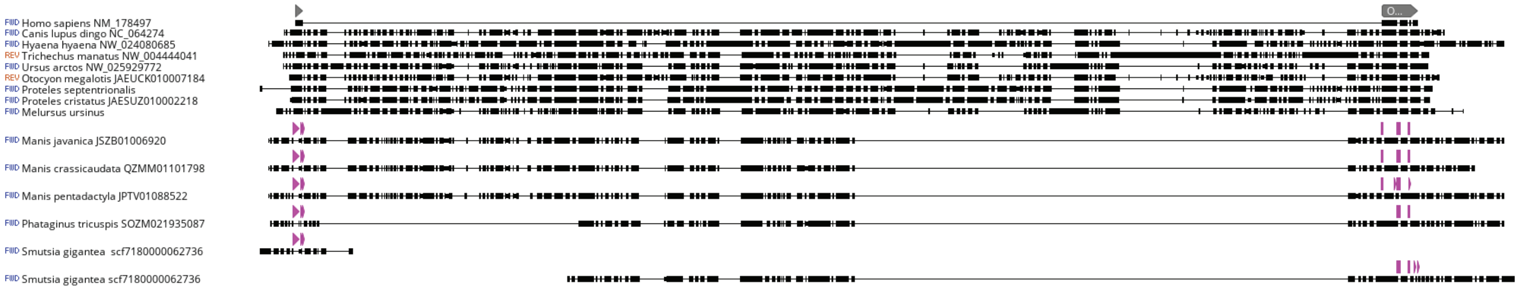

Ostentoria *TAS1R3*

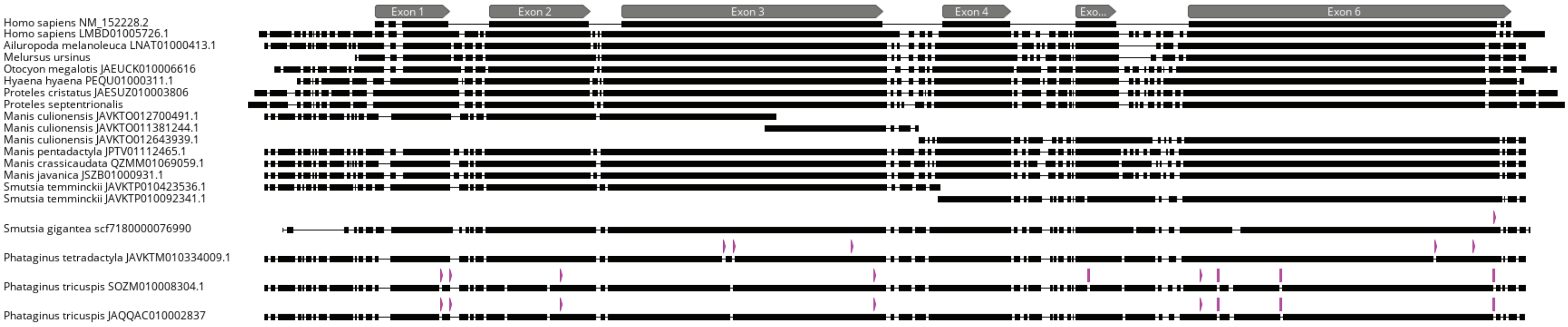

Pholidota *MYH16*

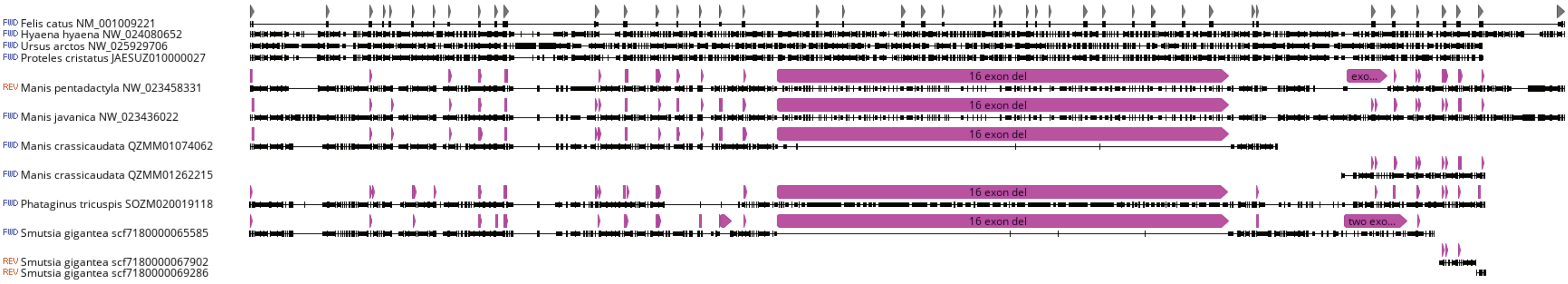

Pholidota *PKD2L1*

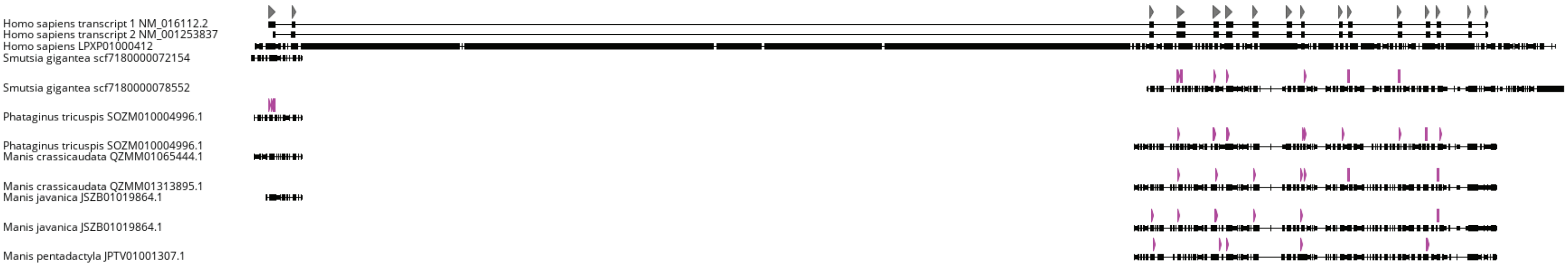

Supplement: msag009_Supplementary_Data [file msag009_supplementary_data.zip › Supplementary Figure S4. Ostentoria ODAM ODAPH TAS1R3 MYH16 PKD2L1.pdf]
