# Supplementary material for: Pseudogenes document protracted parallel regression of oral anatomy in myrmecophagous mammals
Source: Mol Biol Evol. 2026 Jan 13;43(2):msag009. doi: 10.1093/molbev/msag009 (PMC12906968; doi:10.1093/molbev/msag009)

Supplementary Figure S5. DNA sequence alignments for ostentorian (Carnivora + Pholidota) *TAS1R1*. Gray annotations indicate coding exons in reference mRNAs. Pink annotations indicate inactivating mutations.

Ostentoria *TAS1R1*

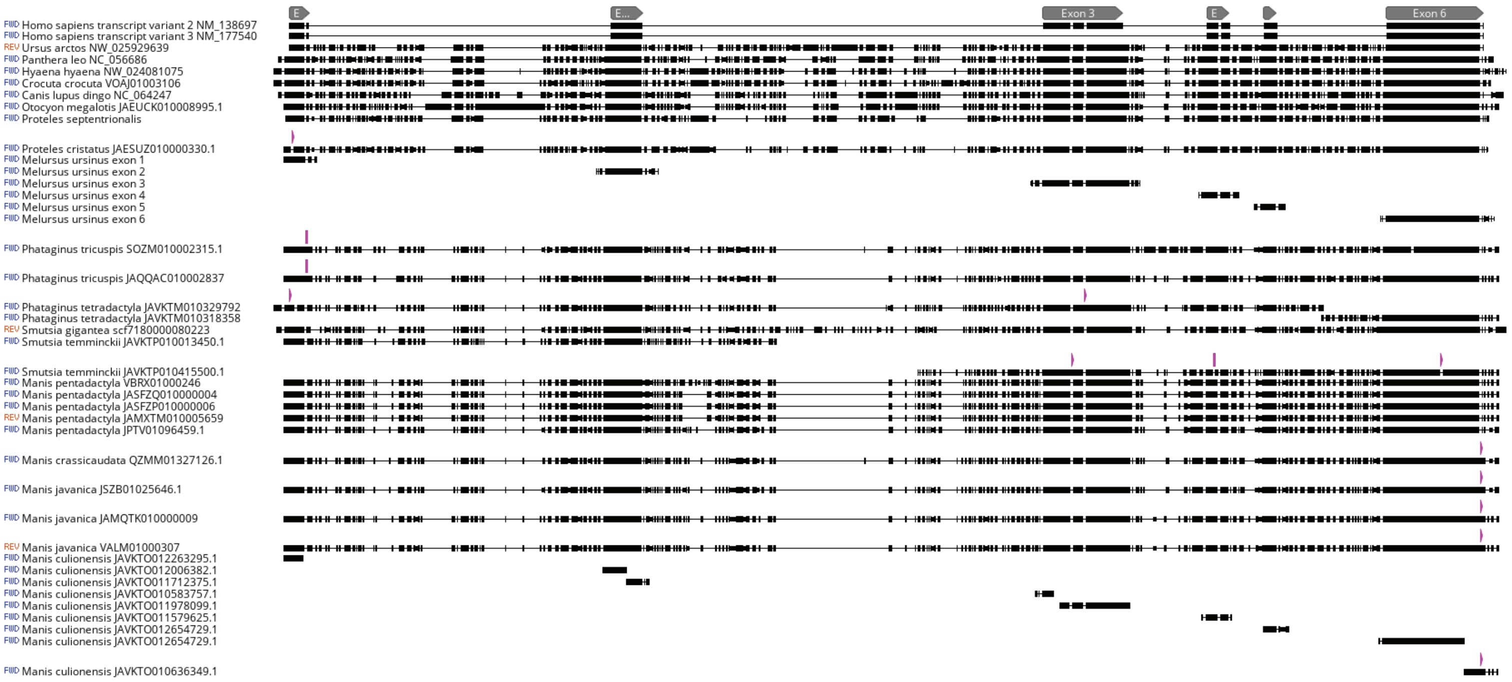

Proteles cristatus NMB12667 *TAS1R1* exon 1 8-bp deletion

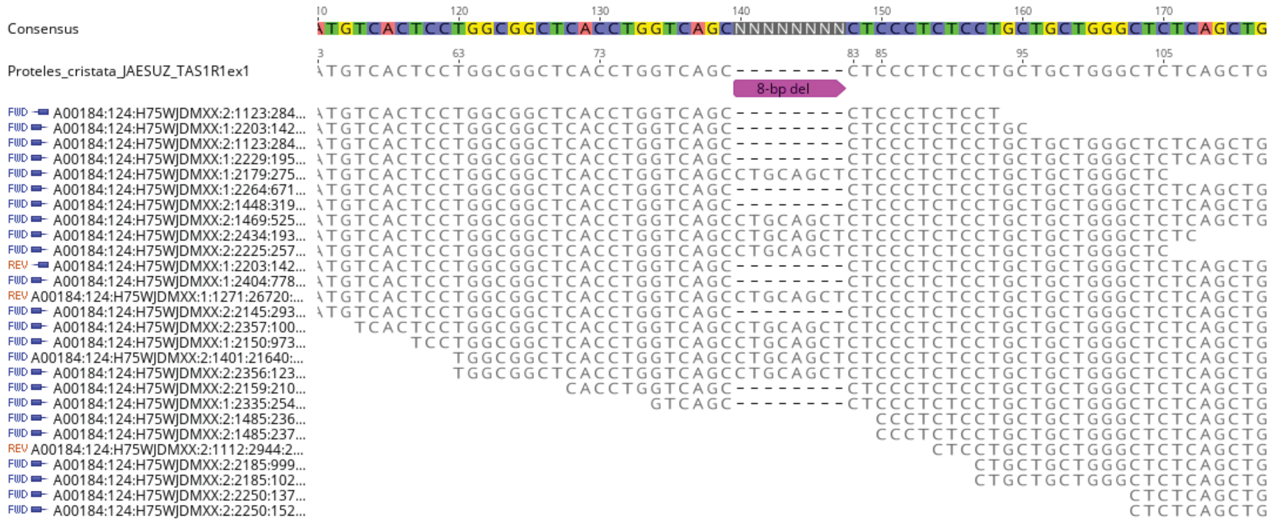

Proteles cristatus NMB12641 *TAS1R1* exon 1 8-bp deletion

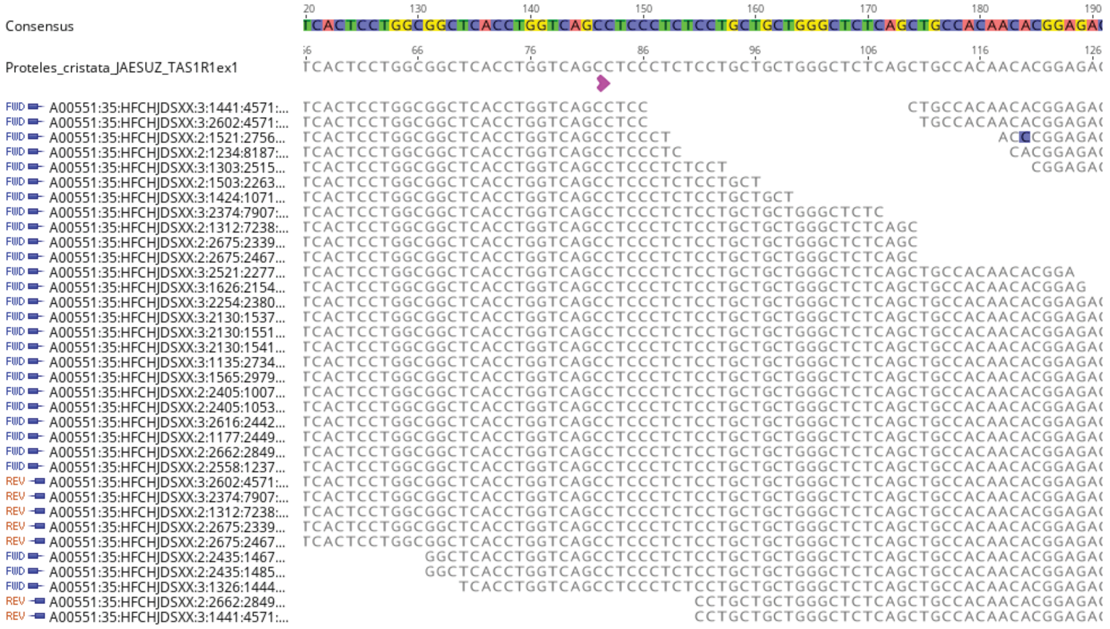

Supplement: msag009_Supplementary_Data [file msag009_supplementary_data.zip › Supplementary Figure S5. Ostentoria TAS1R1.pdf]
