# Supplementary material for: Pseudogenes document protracted parallel regression of oral anatomy in myrmecophagous mammals
Source: Mol Biol Evol. 2026 Jan 13;43(2):msag009. doi: 10.1093/molbev/msag009 (PMC12906968; doi:10.1093/molbev/msag009)

Supplementary Figure S9. DNA sequence alignments for aardvark genes. Gray annotations indicate coding exons in reference mRNAs. Pink annotations indicate inactivating mutations.

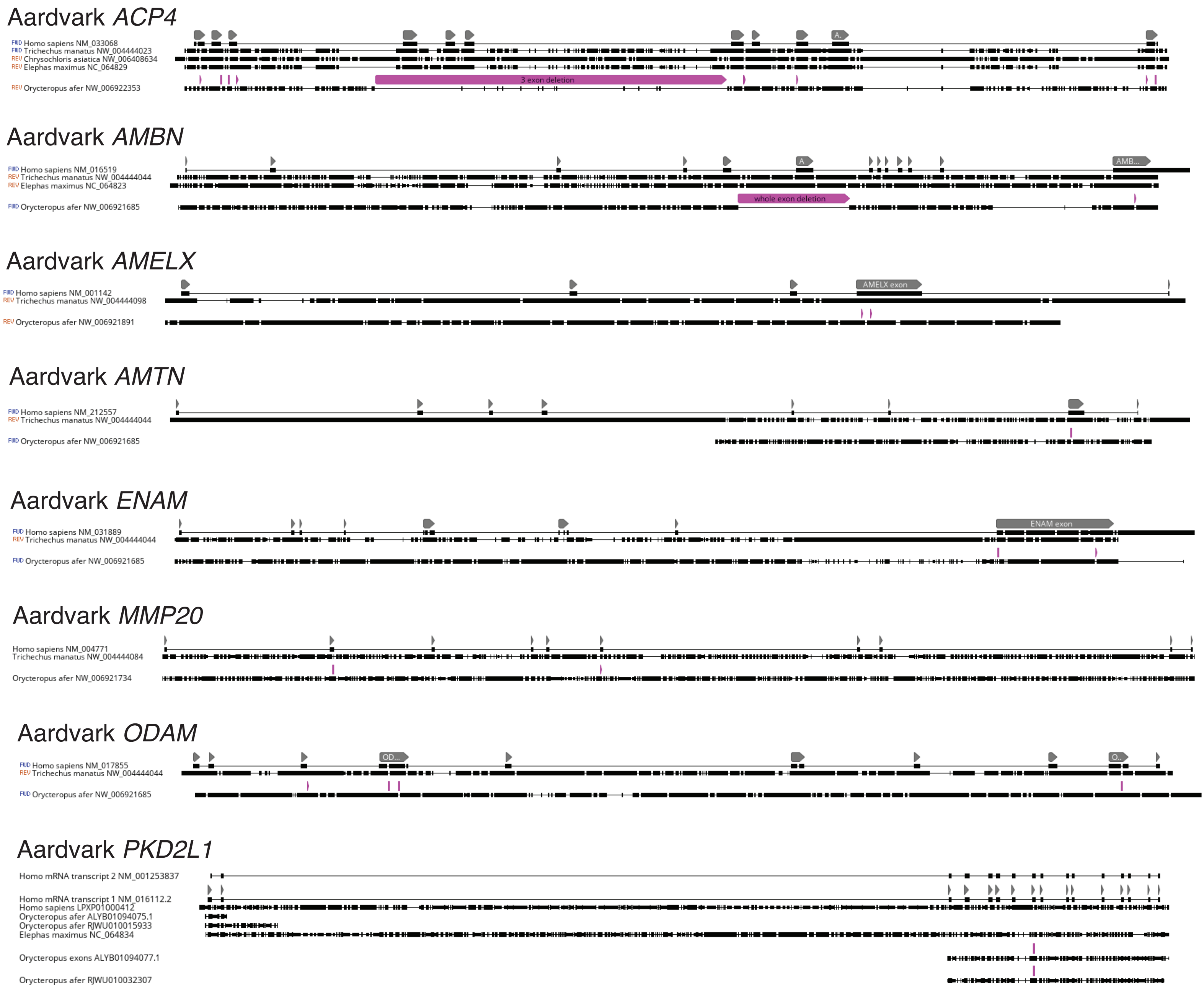

Supplement: msag009_Supplementary_Data [file msag009_supplementary_data.zip › Supplementary Figure S9. Aardvark Mutations.pdf]
