# Supplementary figures and images for: Pseudogenes document protracted parallel regression of oral anatomy in myrmecophagous mammals
Source: Mol Biol Evol. 2026 Jan 13;43(2):msag009. doi: 10.1093/molbev/msag009 (PMC12906968; doi:10.1093/molbev/msag009)

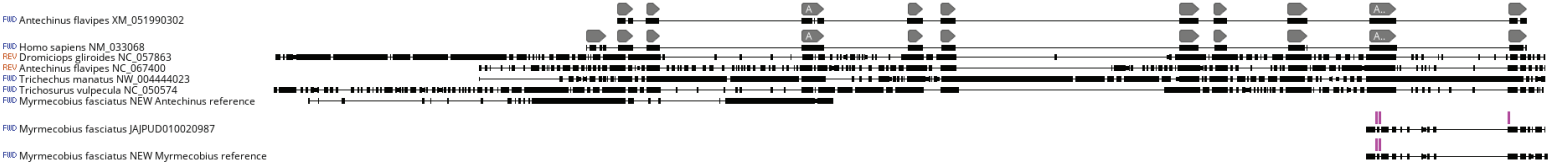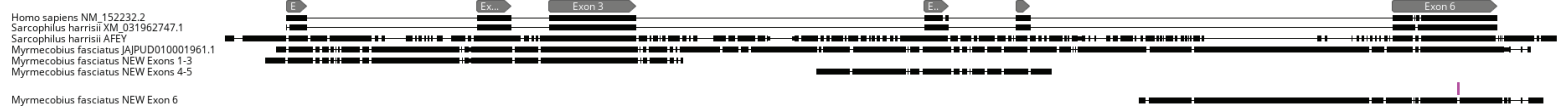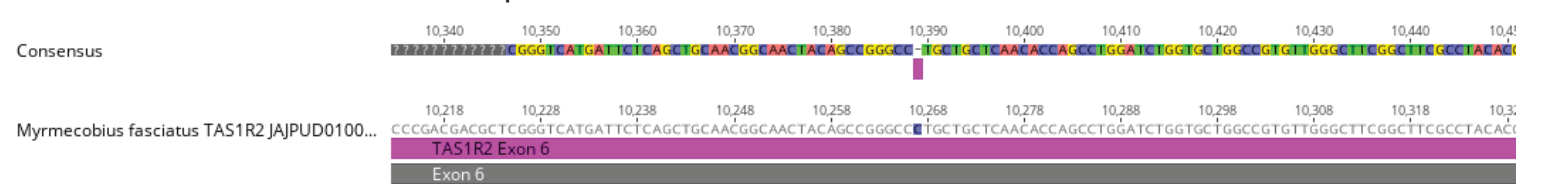[illegible]

Supplement: msag009_Supplementary_Data [file msag009_supplementary_data.zip › Supplementary Figure S10. Numbat ACP4 TAS1R2.pdf]

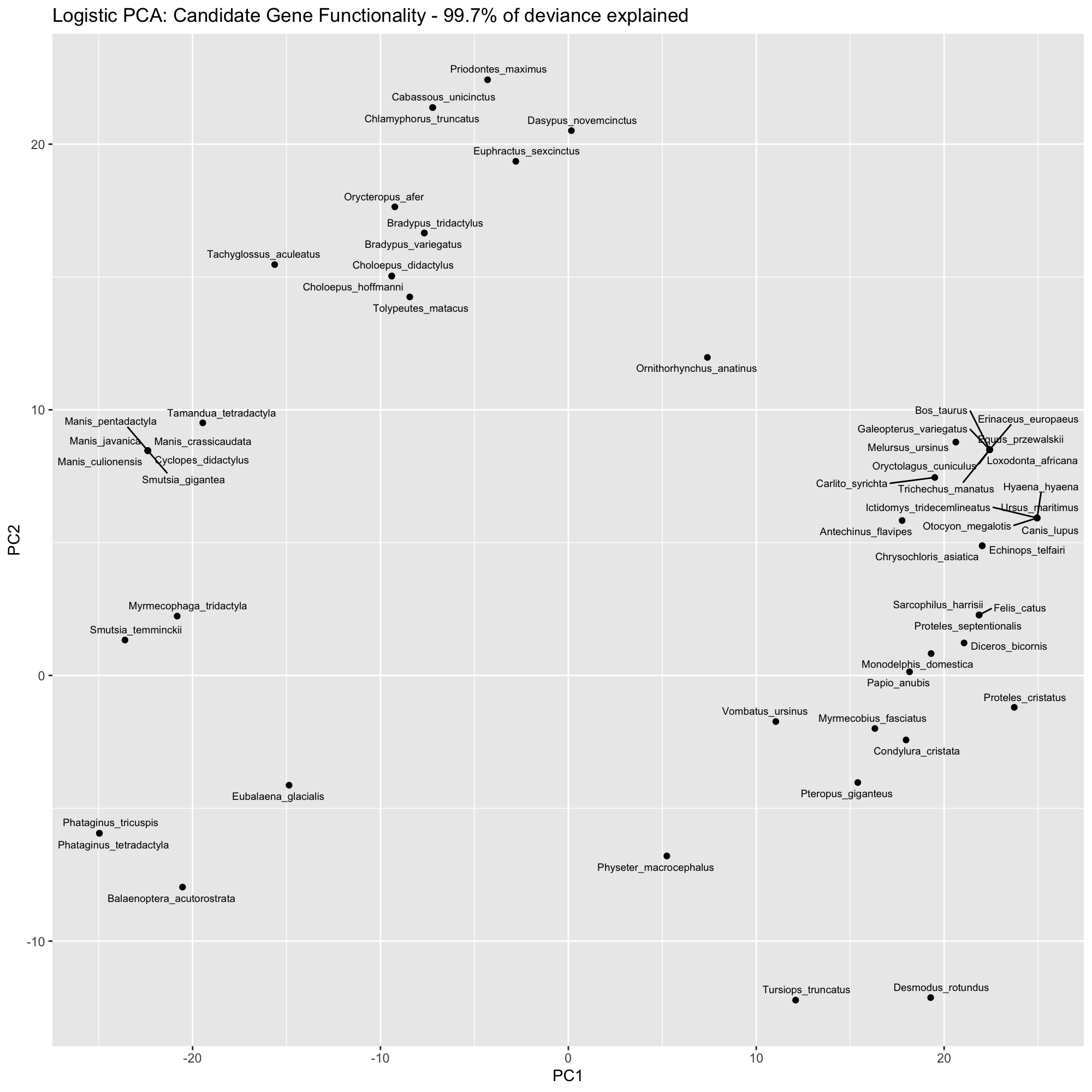

Supplement: msag009_Supplementary_Data [file msag009_supplementary_data.zip › Supplementary Figure S13. Logistic PCA Plot.png]

Comparing OLS and PGLS Regression Fits (Point Size by Count)

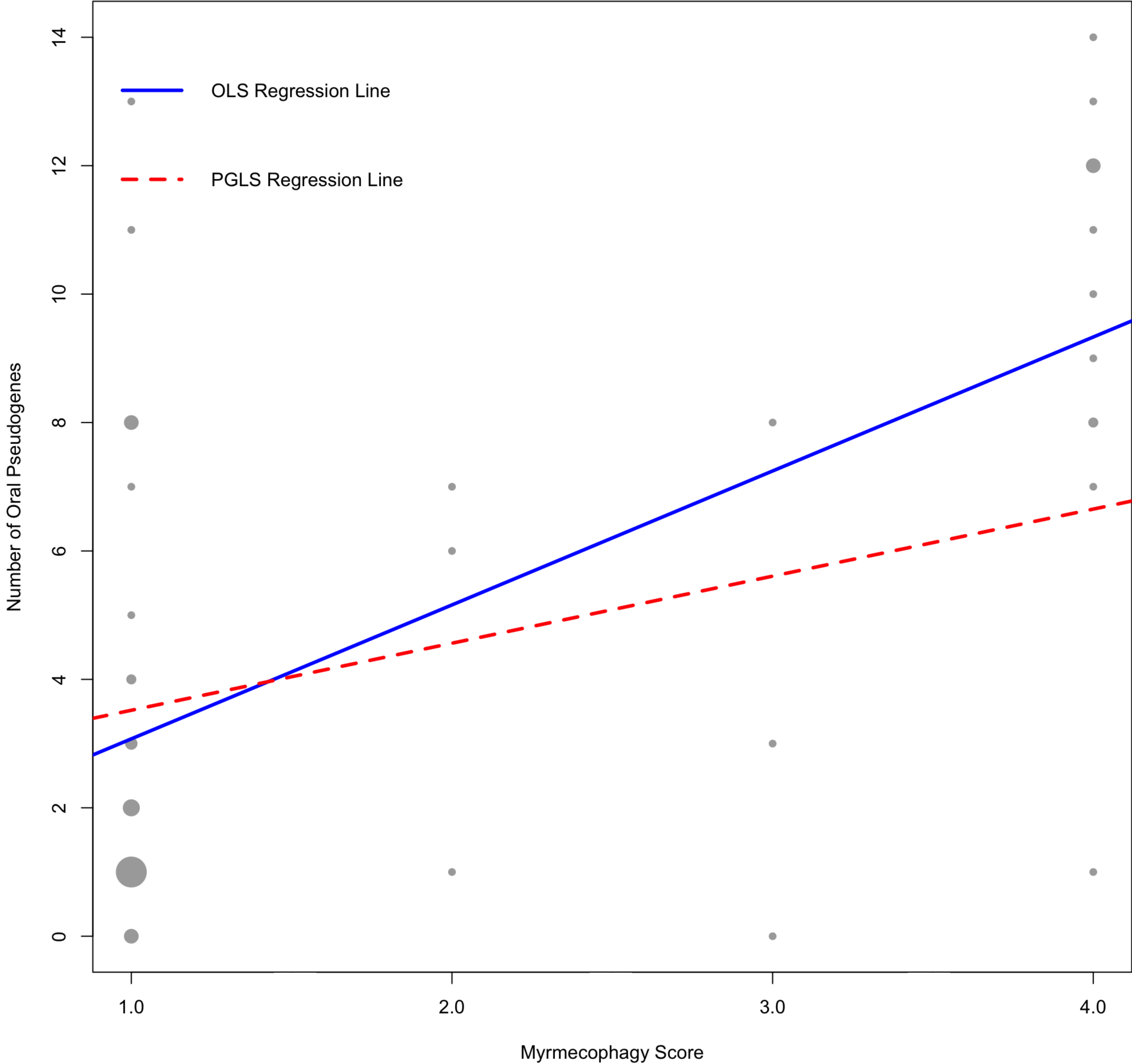

Supplement: msag009_Supplementary_Data [file msag009_supplementary_data.zip › Supplementary Figure S14. PGLS Plot.pdf]
